# Cricopharyngeal bar

**DOI:** 10.11604/pamj.2017.27.288.13296

**Published:** 2017-08-23

**Authors:** Suresh Chander Sharma, Pirabu Sakthivel

**Affiliations:** 1 Department of Otorhinolaryngology & Head and Neck Surgery, All India Institute of Medical Sciences, New Delhi, India

**Keywords:** Cervical dysphagia, cricopharyngeal spasm, myotomy, balloon dilation, botulinu

## Image in medicine

A 65 year gentleman had complaints of difficulty in swallowing primarily for solids along with foreign body sensation in throat for six months duration. Patient also had to do multiple attempts at swallowing the bolus. Occasionally patient also had episodes of coughing during swallowing of liquids. Patient had no loss of weight, fever and hematemesis. Barium swallow revealed narrowing at level of upper esophageal sphincter with prenarrowing dilation and residual contrast (A). On lateral view, a posterior, bar-like protrusion at the level of C5-C6 was seen depicting the cricopharyngeus muscle spasm known as "Cricopharyngeal bar"(B). The patient underwent dilatation with 20mm diameter balloon catheter at distension pressure of 2 atm for 5 minutes and was symptomatically much improved. Failure of the cricopharyngeus muscle to relax at appropriate time during swallowing in the absence of other motor abnormalities results in Primary Cricoparyngeal Dysphagia [PCD]. PCD is rare, occurring mainly in elderly individuals with gastro oesophageal reflux. Cervical block dysphagia is classical symptom which is often accompanied by regurgitation, coughing, choking and aspiration, which may lead to pneumonia. Careful clinical history along with physical examination, radiographic swallowing study and endoscopy with biopsy are necessary. Various treatment options include dilatation, cricopharyngeal myotomy and botulinum toxin injection. Since balloon dilatation is a safe and effective therapeutic procedure, an initial trial of dilatation is probably warranted and other modes of treatment should be recommended for those patients who do not respond to several attempts at dilatation.

**Figure 1:**
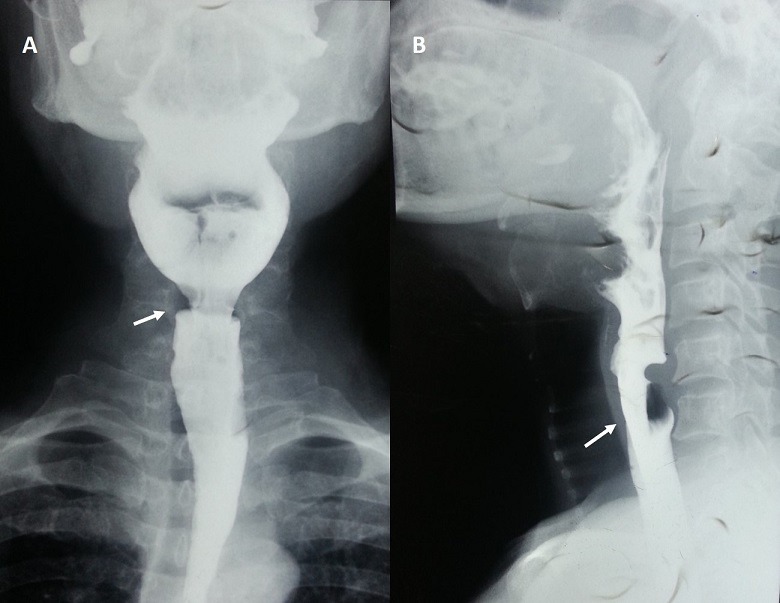
(A) barium swallow AP view revealing narrowing at level of upper esophageal sphincter; (B) lateral view showing a posterior, bar-like protrusion at C5-C6 level-"cricopharyngeal bar"

